# The Modern Use of an Ancient Plant: Exploring the Antioxidant and Nutraceutical Potential of the Maltese Mushroom (*Cynomorium Coccineum* L.)

**DOI:** 10.3390/antiox8080289

**Published:** 2019-08-07

**Authors:** Paolo Zucca, Sidonie Bellot, Antonio Rescigno

**Affiliations:** 1 Department of Biomedical Sciences, University of Cagliari, 09042 Monserrato (CA), Italy; 2 Jodrell Laboratory, Royal Botanic Gardens Kew, Richmond TW9 3DS, UK

**Keywords:** Maltese mushroom, *Cynomorium coccineum*, antioxidant, ethnopharmacology, natural remedy

## Abstract

In the continuous scientific search for new safe and effective drugs, there has recently been a rediscovery of natural substances as a potential reservoir of innovative therapeutic solutions for human health, with the prospect of integrating with and sometimes replacing conventional drugs. *Cynomorium coccineum* subsp. *coccineum* is a holoparasitic plant well known in ethnopharmacology, although its current use as a curative remedy is reported only in some ethnic groups of North Africa and the Arabian Peninsula. Often known as ‘Maltese mushroom’ due to its unique appearance and the absence of chlorophyll, *C. coccineum* is present in almost all of the Mediterranean Basin. It is only recently that a few research groups have begun to look for confirmation of some of its traditional uses to highlight previously unknown biological activities. Here, we review the recent scientific findings on the plant’s phytochemistry and the most significant descriptions of some of its antioxidant and biological activities (antimicrobial, anticancer, pro-erectile, and anti-tyrosinase enzyme) both in vivo and in vitro. Some of these may be promising from the perspective of food and cosmetic formulations. The purpose of this review is to provide an initial impetus to those who, in the foreseeable future, will want to increase the knowledge and possible applications of this plant full of history, charm, and mystery.

## 1. Introduction

The search for new bioactive compounds from the plant kingdom is increasingly gaining the interest of scientific community. In fact, nutraceutical formulations could help in preventing a large number of the diet-associated chronic diseases rapidly emerging in Western countries, including cancer, type 2 diabetes, obesity, and various inflammatory conditions [1]. International agencies strongly encourage the development of high quality, plant-based natural preparations to face such conditions, since this approach is supposed to present fewer adverse effects and be less expensive than common synthetic drugs [1]. The cultivation of such plants, especially if they are endemic, can represent a considerable source of income in developing countries. Similarly, the discovery of natural remedies has also gained a lot of attention in recent decades in the cosmetic sector, even if, in some cases, modern science had not yet confirmed the traditional uses [2].

*Cynomorium coccineum* is a typical example of a plant well known in ethnopharmacology but almost completely forgotten, as ancient folk knowledge has often tended to disappear in recent generations. Some authors hypothesize that even a quote in the Holy Bible may refer to *Cynomorium* [3]. However, it is only recently that a few research groups [4,5,6,7] have begun to look for confirmation of some of its traditional uses and to discover new biological activities.

An explanation for the lack of scientific knowledge we have of *C. coccineum* is that it grows in areas that are usually sparsely populated (deserts, rocky soils, salt marshes).

*C. coccineum* subsp. *coccineum* is present throughout almost the entire Mediterranean Basin, North Africa, and on the Arabic peninsula up to Western China. A second subspecies, *C. coccineum* subsp. *songaricum* (Rupr.) J.Léonard, is present only in Asia, further east than *C. coccineum* subsp. *coccineum* (including the Altay region, Inner Mongolia, Kazakhstan, Kirgizstan, Mongolia, Tadzhikistan, Turkmenistan, Uzbekistan, and the Xinjiang region) [8]. The *C. coccineum* subsp. *songaricum* is widely used in traditional Chinese medicine, and a large number of products containing this herb are marketed [9]. Despite such a widespread use, only one review has been published on this subspecies [9], whereas there has not been any summarizing article about *C. coccineum* subsp. *coccineum*. This review aims to fill this gap by providing an overview of what is known about the chemical content and biological activity of *C. coccineum* subsp. *coccineum*.

## 2. Botany and Folk Medicine

Multiple articles have been published about the taxonomic status and the phylogenetic placement of *C. coccineum* subsp. *coccineum* and *C. coccineum* subsp. *songaricum* [10,11]. For the sake of clarity and to be consistent with the recent phytochemical literature on *Cynomorium*, in the present review, *C. coccineum* subsp. *coccineum* will be referred to as *C. coccineum*, and *C. coccineum* subsp. *songaricum* as *C. songaricum*.

**Figure 1:**
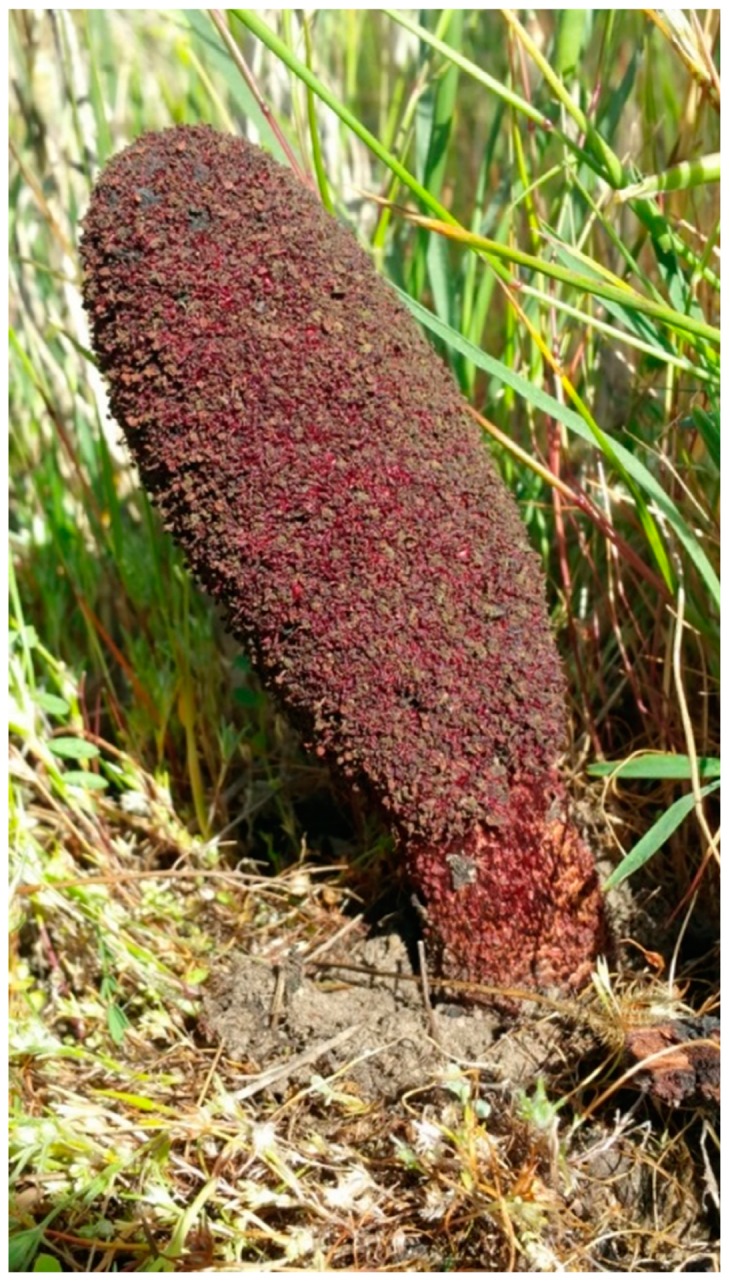
A picture of the aerial part of *Cynomorium coccineum* growing close to a salt marsh (Sardinia, Italy).

*C. coccineum* is a holoparasitic plant, meaning that it does not perform photosynthesis and completely lacks chlorophyll, and therefore it is totally dependent on its host to obtain nutrients. This suggests a different metabolism and metabolic profile from photosynthetic plants, further increasing the scientific interest in this plant and other holoparasites. *C. coccineum* is an herbaceous plant presenting an approximately 4–10 cm high, intensely red-brown inflorescence during the flowering period (Figure 1), which is the only time when this species is visible above ground. It grows in sandy and rocky soils, usually in desert or subdesert habitats, forming haustorial connections with several plants, including Amaranthaceae (such as *Atriplex* or *Salsola*), Asteraceae, Cistaceae, Fabaceae, Frankeniaceae, Plumbaginaceae (*Limonium*), and Tamaricaceae (*Tamarix*).

The stem is covered by scale-like, reddish leaves that do not bear stomata. These scales become scarce towards the inflorescence, which itself comprises hundreds of reddish staminate, carpellate, and, in a lower number, bisexual flowers. *C. coccineum* is, therefore, most often monoecious. The staminate flower is made of (1–3) 4–6 (7–8) spatulate perianth parts forming a whorl or irregularly spiral arranged below a single stamen. The stamen filament nests in a longitudinal groove on the inner side of a semi-cylindrical or wedge-shaped pistillode with a truncated or notched apex. The carpellate flower is mostly made of a carpel with an elongated, grooved style, and its perianth is reduced to 1–8 small, free papillae at the summit of the ovary or along its sides.

According to Léonard (1986) [12], *C. songaricum* has male tepals that can be as wide as 1.5 cm, and the pistillode accompanying the anther filament is white, while in *C. coccineum*, male tepals are never wider than 0.8 cm and the pistillode accompanying the anther filament is reddish. The pollen of *Cynomorium* is tricolporate [13]. The red color of the scales and flowers is due to flavonoids (*vide infra*) [14]. The almost cylindrical shape of the plant, combined with the absence of green color and its sprouting directly from the ground without any clearly visible leaves or stem, resulted in it being confused with a mushroom for a long time. In fact, *C. coccineum* is known under several vernacular names reflecting this confusion, including *Fungus Typhoides*, *Fungus coccineus*, and *Fungus gozitanum*, and also Maltese mushroom. The appellation ‘Maltese’ comes from a Maltese growing site of the plant (known as the Fungus Rock) off the coast of Gozo (Maltese archipelago), where the Knights of the Malta Medieval Order protected and used *C. coccineum* to cure the bleeding wounds of knights struck in battle, and dysentery, rather frequent at that time because of the precarious hygienic conditions. *C. coccineum* is also known in Arab countries as Tharthut, Tarthoorth, or Zobb el Ard.

Ibn Sina (980–1037 CE), also known in the west as Avicenna, was a Persian Muslim polymath who is regarded as one of the most significant physicians of the Islamic Golden Age [15]. Avicenna mentions ‘Maltese mushroom’ [Cynomorium] as part of an excretory ointment,’ which ‘relaxes the bowels and is useful in treating chronic diarrhea’ [16]. For an exhaustive description of medical prescriptions over the centuries, see the admirable work of Lanfranco (1960) [17]. The ancient uses of *C. coccineum* in traditional medicine are reported in Table 1.

The scientific name *Cynomorium* reflects the resemblance of the plant to a dog’s penis (in ancient Greek, “*cyno*”). Due to this particular shape, many traditional uses of the plant involve the reproductive system [19,24], in accordance to the *Signatura Rerum*. The *Signatura Rerum* (Doctrine of Signature) is an ancient document, formulated for the most part by Paracelsus, that describes the phenomenon whereby a plant, or a part of it, serves to heal precisely the organ of the human body from which it takes the shape. For instance, if a plant has a red juice, it will be expected to ‘cure the blood,’ or the walnut, having a brain shape, will be used to cure pathologies. In a similar way, in some cultures, *C. coccineum*, with its phallic appearance, is considered an aphrodisiac for males, and in others, for females [24]. The plant is also believed to affect fertility, show anti-hemorrhoidal properties, and regulate menstrual disorders [19]. These popular beliefs have led some researchers to study *C. coccineum* properties in more detail with regard to its effects on the reproductive system. 

## 3. Phytochemistry

Despite a long history in traditional medicine, the phytochemistry of *C. coccineum* is still largely unknown and has been the subject of a very limited number of studies so far (summarized in Table 2). More phytochemical data are available for *C. songaricum*, from which over 40 different chemicals have been identified, including phenolics (i.e., flavonoids, phloroglucinol derivatives, phenylpropanoids), steroids, organic acids, terpenoids, and sugars [9].

Cyanidin 3-*O*-glucoside was identified as the pigment component of an acidified hydroalcoholic extract from the red inflorescence of the plant collected in Spain [14], as well as from water extracts obtained from Sardinian samples [7], while Harraz et al. (1996) additionally identified a minor presence (8% of total anthocyanins) of cyanidin 3-*O*-rhamnosylglucoside [25].

These anthocyanins are confined to the red external layer of small flowers and are almost absent in the colorless stem and internal part of the plant. So far, no anthocyanins have been described for *C. songaricum* [9,26]. In the Asian subspecies, in fact, other types of flavonoids (i.e., flavan-3-ols, flavanones, and flavones) have been detected. The similar color of the two species suggests that some colored chemicals remain to be identified in *C. songaricum*. 

In general, several studies have reported that spectrophotometrically quantified flavonoids account for a significant portion (about 25–50%) of the total phenolic content of the plant [7,27,28]. Some of these could possibly be procyanidin oligomers, as suggested by Zucca and co-workers [7], but the authors failed to identify the chemicals.

Recently, Jabli et al. (2018) highlighted the coloring power of these anthocyanins for textile materials, suggesting a possible use as textile dyes [29]. This could be a remarkable use of *C. coccineum*, taking into account all the environmental concerns regarding the chemical durability and toxicity of synthetic dyes [30].

As a principal component, gallic acid has been identified in almost equal distribution between the flowers and the colorless internal part of the plant by means of HPLC [7]. The same authors highlighted unidentified HPLC peaks showing similarities with gallic acid derivatives (possibly gallotannins). Gallic acid was quantified at around 4 mg·g^−1^ dry extract, and could explain some biological properties of *C. coccineum* (see below). For example, gallic acid has been reported to possess wound healing properties [31] and constipating properties. Similar properties have been reported for fruits of other plants; for example, *Terminalia chebula* fruits, used as an herbal remedy for diarrhea in traditional Chinese medicine, also contain tannins, including gallic acid and related glycoside derivatives, as major components of the ethyl acetate fraction [32].

The presence of tannins and flavonoids was also recently confirmed in samples from Saudi Arabia, using spectrophotometric and qualitative assays [5]. However, even in this case, no chromatographic identification was attempted. Using the same approach, the presence of glycosides, anthraquinones, saponins, alkaloids, and terpenes has been highlighted in extracts with different polarities (*n*-butanol, water, aqueous methanol, and hexane) [5].

In a recent work, Ben Attia et al. (2018) compared the chemical compositions of *C. coccineum* plants from Sardinia and Tunisia, describing a differential chemical profile, possibly due to the climatic conditions (more arid in the Tunisian desert, and temperate in the Mediterranean basin). The phenolics, in fact, were differently distributed among solvents at various polarities [33]. Additionally, these authors used ^1^H-NMR to identify several amino acids (including proline, glutamine, valine, threonine, alanine, and asparagine) and mono-, bi-, and tricarboxylic organic acids (i.e., acetate, formate, and several common intermediates of biochemical pathways such as citrate, fumarate, malate, malonate, and succinate) in Sardinian and Tunisian samples. Moreover, sugars (i.e., β-glucose, α-glucose, fructose, and sucrose), as well as the two quaternary ammonium salts choline and betaine, were quantified in the same study. It resulted that betaine was significantly more concentrated in the samples from Tunisia. A correlation between betaine accumulation and abiotic stress (i.e., the arid climate) has been suggested, since betaine is known to be involved in osmoregulation and osmoprotection, being associated with environmental stresses such as salinity and extreme temperature [35,36].

In a series of papers, the lipid profile of Sardinian *C. coccineum* Supercritical Fluid Extract (SFE) oil has been elucidated [34], showing an almost 1:1:1 concentration of saturated fatty acids (SFA, mainly palmitic acid 16:0 and stearic acid 18:0), monounsaturated fatty acids (MUFA, mainly oleic acid 18:1 n-9), and polyunsaturated fatty acids (PUFA) [28,37]. The same authors confirmed this pattern in a Tunisian sample [33], suggesting a promising nutraceutical source of functional and beneficial compounds (for example, about 11% of the SFE oils was 18:3 n-3). However, different accessions (also from the same geographical area) showed a high variability not only in the total quantity, but also in the oil composition [28,34], probably due to different annual weather fluctuations or small differences in the collection/extraction procedures. The composition of fatty acids seems to be quite similar to that of *C. songaricum* [38], with palmitic and oleic acid being the quantitatively most represented.

The compatibility of *C. coccineum* formulations with the human diet was shown by the ‘Nutritional Facts Label’ (reporting the quantity of the three macronutrients), which established that 100 g of dried whole plant contained around 45 g carbohydrates, 9 g proteins, and approximately 1 g fatty acids, while total dietary fiber accounted for about 28 g [28]. These findings seem to explain the use of *C. coccineum* as an emergency food in time of famine, of which a few examples can be found in the literature [39].

## 4. Antioxidant Activity

Reactive oxygen species (ROS) are unavoidable oxidized sub-products of cellular aerobic metabolism [40]. This results in a paradox where, while most complex organisms require O_2_ for their existence, at the same time, oxygen is a highly reactive molecule that damages them by producing ROS [41]. In recent decades, oxidative stress has been implicated in several degenerative processes, diseases, and syndromes, including: carcinogenesis, mutagenesis, impairment of fertility, atherosclerosis and cardiovascular disease, acute and chronic inflammatory diseases, oxidative photodegradation of ocular tissues, central nervous system disorders, and a wide range of age-related disorders (see Reference [41] and references therein). Free radicals can also originate from prolonged exposure to UV light, cigarette smoke, and air pollution [42]. 

Living organisms counteract the activity of free radicals through endogenous, enzyme-based antioxidant mechanisms (e.g., enzymes such as SOD and catalase) or mechanisms involving low molecular weight compounds (such as reduced glutathione) [43]. Although the protective role of these molecules is very important, they are not completely effective in counteracting oxidative stress damage, and the introduction through the diet of exogenous antioxidant substances (vitamins, carotenoids, polyphenols, and anthocyanins) is strongly advised [44,45]. Currently, great attention is being focused on the possible protective value of a wide variety of plant-derived antioxidant compounds, particularly those from fruits and vegetables. In plants, antioxidant molecules are produced as secondary metabolites and play a protective role against stressful conditions. The main classes of these compounds are phenolic acids, flavonoids, flavanols, and anthocyanins [46]. The many beneficial effects on human health attributed to these compounds have given rise to a growing interest in the search for plant species with high antioxidant content and relevant biological activities (such as antimicrobial, anti-inflammatory, and anti-melanogenic activities).

Comparing antioxidant power is quite difficult, even inside a single plant species, as there is no uniformity in the use of extraction techniques, in the collection, conservation, and treatment methods of the raw material, or in the choice of the antioxidant assays used. Therefore, in this section we have tried to make a comparison and discuss the experimental data and the most widely used methods [47] reported by different authors (and summarized in Table 3).

These methods (transfer of electrons or hydrogen atoms, and determination of flavonoids and phenolics) generally provide a total estimate of the antioxidant power of a sample. Rached et al. (2010) related the total antioxidant power to the number of potentially antioxidant phytochemical compounds in aqueous and ethanol extracts of *C. coccineum* grown in Algeria by means of an antioxidant activity test by TLC bioautography. This test gave several anti-radical spots [27].

The authors compared the antioxidant power of *C. coccineum* extracts with extracts from 52 different Algerian plants. Examination of Table 3 shows that *C. coccineum* extracts had excellent antioxidant characteristics. The ethanol extract, in particular, possessed one of the highest contents in total phenols and flavonoids, and a value of [DPPH•] radical scavenging activity comparable to that possessed by BHT (butylated hydroxytoluene) a synthetic antioxidant widely used in the agri-food industry. Such a value was in line with that reported for a butanol extract of *C. coccineum* collected in Saudi Arabia. In fact, Al-humaidi (2016) showed an IC_50_ value of 5.6 μg·mL^−1^: a value very close to that of the ascorbic acid used as a control (Table 3) [5]. In this work, after removing the lipid material by treatment of dried *C. coccineum* specimens with petroleum ether, a first extraction with methanol (raw extract) was performed, followed by other extraction phases with different extracting phases (aqueous methanol, butanol, water, and hexane). This double and sequential extraction procedure probably explains why the water extract of this study did not contain flavonoids, unlike what was reported in the work of Rached (2010). The 2,2′-azino-bis(3-ethylbenzothiazoline-6-sulphonic acid) (ABTS) assay gave similar results for the various types of extract. The estimated IC_50_ for [ABTS•^+^] radical scavenging activity was maximal for the butanol extract, with values very close to those measured for ascorbic acid and the tocopherol used as a control. 

Specimens of *C. coccineum* grown and harvested in Sardinia (Arborea, Italy) showed a slightly lower antioxidant power for the whole plant (Table 3). The authors of this study [7], however, measured the antioxidant properties of the methanolic extract of *C. coccineum* using different biochemical assays. They showed that this extract (5 µg) was able to inhibit the degradation of cholesterol in oxysterol by 70% in an in vitro model system. The protective antioxidant effect of the methanol extract was also exerted against Cu^2+^-mediated degradation of liposomal unsaturated fatty acids in vitro.

A confirmation of the fact that the location and environmental conditions of plant growth can also affect the plants’ antioxidant power comes from a recent study. In the same laboratory, the same methods of extraction and analysis of *C. coccineum* specimens grown and collected in geographically distinct places (Italy and Tunisia) with very different climatic characteristics [33] were used. The *C. coccineum* specimens were collected in a desert region in south-eastern Tunisia and in an area near the coast of Sardinia. These samples were macerated in sequence with increasing polarity solvents (n-hexane, chloroform, ethyl acetate, acetone, methanol, and water). The residual material coming from the maceration with the first solvent constituted the material that was extracted with the subsequent solvent. The peculiar extraction procedure described does not allow a comparison with the results reported in Table 3, however, it allows some considerations. The five extracts thus obtained showed profound differences in terms of antioxidant properties. The differences depended on the type of extracting solvent. The highest antioxidant activity was retrieved in both acetone extracts, which also were the richest in polyphenols. 

The specimens collected in the arid climatic zone were richer in anthocyanins, while the samples from Sardinia showed a higher content of total phenolics. The greater presence of anthocyanins in the Tunisian samples (arid climate) confirmed the role that these compounds play in drought stress. Overall, total antioxidant activity did not seem to be too different between the two samples, despite markedly different phenolic profiles. 

The results shown above demonstrate that *C. coccineum* extracts have a remarkable antioxidant activity, as shown by the comparisons with the antioxidant activity of ascorbic acid, alpha-tocopherol, and BHT. In some cases, substantial differences have been reported in relation to the extraction methods and solvents used. It is known that the process of extracting antioxidant molecules from plant material is influenced by various factors, such as quantity, chemical nature, extraction methods, the presence of interfering substances, etc. [48]. Therefore, the extraction of antioxidant compounds from plant materials generally requires a set of different steps to ensure the removal of unwanted substances. In the case of *C. coccineum*, extractions with polar organic solvents (MetOH, EtOH, and ButOH) are those that provide a greater yield. However, water extracts showed significant antioxidant power, which, combined with the other biological activities described below, opens the way to food applications for *C. coccineum*-based formulations.

Unfortunately, to our knowledge, no in vivo studies to date have confirmed the ability of this plant to counteract oxidative stress. This is a great lack, hindering the extension of the potential applications of *C. coccineum*-based formulations.

## 5. Biological Activity

UV radiation or drought stress stimulate the synthesis of secondary metabolites that are responsible for different types of biological activity. Growing in such harsh environmental conditions, *C. coccineum* is expected to produce metabolites with biological activity (summarized in Table 4).

### 5.1. Inhibition of Cancer Cell Proliferation

It has long been known that diets rich in fruit and vegetables are associated with a reduced risk of cancer, and phytochemicals, especially numerous phenolic compounds, capable of exerting antitumor activity have been identified in many plants [49]. Various mechanisms have been suggested to explain the beneficial effects of fruit and vegetable consumption, for example, promotion of the detoxification of carcinogens, inhibition of carcinogenic activation, scavenging of free radical species, and inhibition of cell proliferation. From this perspective, some authors have investigated the ability of *C. coccineum* extracts to show antiproliferative activity against cancer cell lines. Rosa and co-workers (2012) used CO_2_ as supercritical fluid to extract the dried aerial part of *C. coccineum*. CO_2_–SFE extraction, performed at 250 bar and 40 °C, gave a colored oil with a strong odor [34]. 

This oil was mainly composed of triacylglycerols and related derivatives, and traces of sterols. The main fatty acids were a mixture of polyunsaturated, monounsaturated, and saturated fatty acids (as already described). This study clearly demonstrated a cytotoxic effect of fixed *C. coccineum* oil on human colon cancer cells (Caco-2) in vitro.

This cytotoxic effect was also tested in the presence of 5-fluorouracil (FU) in vitro [37]. FU is a well-known anticancer drug which acts by preventing the duplication of DNA, the synthesis of RNA, and cell division. Interestingly, at non-cytotoxic concentrations of fixed *C. coccineum* oil, the effect of FU was enhanced. 

In fact, 2 hours of pre-treatment with this oil increased the cytotoxicity of FU, and significantly decreased (30%) cell viability. The growth inhibition properties of the oil were probably due to changes in the lipid composition of the Caco-2 cell membrane. The oil lipids altered the structure and fluidity of the cell membrane, and, probably, the signaling transduction pathway mediated by lipids [34].

Inhibition of metastatic cell growth has also been demonstrated using aqueous *C. coccineum* extracts. Zucca et al. [28] found that extracts of the whole plant were able to reduce the viability of B16F10 murine melanoma cells, a highly metastatic cancer cell line that is commonly used as a target to screen antitumor agents. B16F10 cell viability decreased by 41% and 68%, compared to the control, when they were treated in vitro with 250 and 500 μg·mL^−1^ of whole plant extract solutions, respectively. It is interesting to notice that the fixed oil was not able to inhibit the growth of B16F10 melanoma cells [37]. This observation suggests that one or more water-soluble substances present in the extract were responsible for the inhibitory effect. This effect could be linked to the presence of large quantities of gallic acid in the water extract [7], as suggested by the fact that gallic acid was recently shown to induce cellular apoptosis in B16F10 melanoma cells [56], probably through a pro-oxidant action of gallate compounds [57].

Antiproliferative properties of *C. coccineum* extracts have been confirmed in recent work [53]. Sdiri et al. (2018) compared the cytotoxicity of three different extracts (water extract, polysaccharide extract, and ethanol extract). The ethanol extract showed a high capacity to decrease the viability of human breast cancer cells (line MDA-MB-231) at very low concentrations in vitro. B16 murine melanoma tumor cells, by contrast, seemed more sensitive to a water extract with an IC_50_ value of about 34 μg·mL^−1^. Interestingly, Sdiri et al. noted that the ethanol extract also inhibited cellular invasion, cell migration, and colony formation of MDA-MB-231 cells. Experiments were also conducted in vivo on mice injected with melanoma tumor cells. Daily administration of water extract (50 mg kg^−1^) significantly prolonged the survival of the mice. This is the first report on the antitumoral effect of *C. coccineum* extract in vivo. These authors believe that there are one or more yet-to-be-identified active molecules in *C. coccineum* that could help in a therapeutic approach aiming to reduce the administration of chemotherapy.

### 5.2. Activity on the Reproductive System

Herba Cynomorii (*C. songaricum*) is included in the Chinese Pharmacopoeia, where it is reported as a remedy for the treatment of impotence, premature ejaculation, and spermatorrhea [9]. *C. coccineum* has been recently studied for its possible effects on the reproductive system. The effect of aqueous extract of *C. coccineum* on the epididymal sperm pattern of rats has been studied by Abd El-Rahman and colleagues [4]. They administered the extract to a group of 10 adult male Wistar rats for two weeks, and the results were compared with a second group of 10 rats. The results, analyzed after the sacrifice of the animals, revealed that the group fed with the extract presented an increase in sperm count, and improved percentage and motility of live sperm. Histological examination of testicular tissue showed a general increase in spermatogenesis and seminiferous tubules filled with sperm in the treated group compared with the control group. The biological effects found suggest a testosterone-like activity, confirming the results of Harraz et al. [25], which showed that *C. coccineum* water extract induced spermatogenesis in immature Wistar rats. Unfortunately, the authors have not attempted to isolate any compound responsible for such activity. 

However, serum testosterone and follicle stimulating hormone (FSH) levels were lower in immature 20 day old male Wistar rats fed with *C. coccineum* water extract for 6 days than in the controls [52]. FSH stimulates testicular growth and enhances the production of an androgen-binding protein by the Sertoli cells, which are a component of the testicular tubule necessary for sustaining the maturing sperm cell. Unlike FSH, the levels of interstitial cell stimulating hormone (ICSH) were higher in treated animals. ICSH, secreted by the anterior pituitary gland, acts by developing the interstitial tissues of the testis and stimulating the secretion of androgenic hormones. The interpretation of these results is rather complex and did not allow the authors to outline a single possible scenario. However, the authors drew the conclusion that the water extract stimulated testicular development and spermatogenesis in immature Wistar rats by directly affecting the seminiferous tubules [52]. 

The influence of *C. coccineum* water extract was also investigated on the reproductive apparatus of female Wistar rats. Immature 17 day old and 25 day old animals were fed with the extract for 6 days [6]. Significant changes in gonadotrophin levels (FSH and LH) coupled with an increase in ovarium weight and folliculogenesis were observed, especially in the 25 day old rats. However, it remains unclear whether these effects are exercised directly on gonadotropins or via the hypothalamus.

Stimulated by the results of the studies carried out on the reproductive apparatus using extracts of *C. coccineum*, Zucca et al. (2016) studied the effect of aqueous extract on penile erection in laboratory animals [28]. Different extracts were administered to adult male Sprague–Dawley rats. The first extract tested, named ES-1, (Extract Solution-1) consisted of the aqueous extract of the whole plant. It appeared as a red solution due to the presence of anthocyanidins from the outer aerial part of the *C. coccineum*. An aliquot of ES-1 was passed through a C18 chromatographic column. The column was washed with 50% MetOH in water. The solution thus eluted was the extract ES-2. It must be emphasized that the ES-2 extract was totally colorless. A subsequent washing of the C_18_ column with 100% MetOH gave the extract ES-3, intensely colored red. The three different extracts were administered to a first group of rats, while a second control group received a similar amount of saline solution. The whole plant extract, ES-1, exhibited pro-erectile activity when administered subcutaneously to adult male rats. It is interesting to note that the pro-erectile activity persisted even after the colored compounds were removed (ES-2 extract), while the colored extract, ES-3, was substantially inactive. It was therefore concluded that the pro-erectile activity was due to one or more non-colored, highly hydrophilic compounds present in the whole plant. The structure of this compound(s) is currently unknown. The effects of the administration of 10 mg of ES-1 were similar to the pro-erectile activity exerted by 20 µg of apomorphine, a dopamine receptor agonist well-known for its ability to induce penile erection in laboratory animals.

### 5.3. Anti-Tyrosinase Activity

Tyrosinase refers to a class of oxidase enzymes involved in two distinct melanin synthesis reactions: (i) the hydroxylation of a monophenol in o-diphenol and (ii) the conversion of an *o*-diphenol into the corresponding *o*-quinone [58,59,60]. Tyrosinase substrates are not limited to mono- and di-phenols, since o-aminophenols and aromatic diamines have also been proven to be substrates of these enzymes [61,62]. The tyrosinases are responsible for the formation of melanin or melanin-like pigment in most living species, and are also involved in the formation of byssus threads [63,64] Enzyme inhibitors are used to prevent hyperpigmentation of the skin, and to prevent browning of fruits, vegetables, and shrimps [65,66,67]. The discovery of new tyrosinase inhibitors is strongly desired due to the general increase in interest in substances of natural origin, to the detriment of substances deriving from chemical synthesis [62,68,69]. From this perspective, it is interesting to highlight the work of Zucca and co-workers (2016), who discovered that water *C. coccineum* extracts were able to inhibit the activity of mushroom tyrosinase, the enzyme commonly used as a model for studies on melanin biosynthesis [28]. All the extracts from the various parts of the plant (WP, whole plant, EL, external layer, and PP, peeled plant) were able to inhibit tyrosinase, although the WP extract exhibited a stronger inhibition than the sum of both its components (EL and PP). A value of IC_50_ = 15.6 μg mL^−1^ suggested that the WP secondary metabolites played a decisive synergistic role in the anti-tyrosinase activity of *C. coccineum*. This finding was supported by the fact that cyanidin-3-*O*-glycoside (the principal anthocyanidin of EL) possessed an IC_50_ value of 23.6 μg mL^−1^. These values were in line with what was obtained from other parasitic non-photosynthetic plants belonging to the genus *Cytinus* [70]. In this way, *C. coccineum* appears to be a promising candidate for the preparation of formulations based on natural substances for the control of browning.

### 5.4. Effects on the Cardiovascular System

One of the few, and perhaps the first, studies in vivo of *C. coccineum* extracts was performed on dogs. In an attempt to identify biologically active water-soluble fractions of the plant, Ikram et al. (1978) found a blood-pressure-lowering activity of the fresh fruit juice. This lowering effect did not occur in extracts with organic solvents. Unfortunately, the authors failed to identify any component to which to attribute this effect [55], and in the following years, to our knowledge, no studies were carried out that could confirm this interesting biological activity of *C. coccineum*.

Ashour et al. (2012) have explored the potential protective effects of methanolic extracts of several Saudi plants against doxorubicin-induced cardiotoxicity in rats [54]. Doxorubicin (DOX) is an anthracycline widely used as a chemotherapeutic in the treatment of several tumors. However, its cardiac toxicity prevents its use at maximum therapeutic doses. Most studies have reported an increased oxidative stress as the major determinant of DOX cardiotoxicity. 

As shown above, *C. coccineum* methanolic extracts have significant antioxidant properties. This could be the reason why pre- and co-treatment with the plant’s methanolic extracts protected against DOX-induced GSH (reduced glutathione) depletion, lipid peroxidation, and elevated activities of serum creatine kinase-MB (CK-MB), a marker used to monitor the health of the heart. However, this extract did not alleviate the histopathological alterations induced by DOX.

### 5.5. Antimicrobial Activity

Several sources report *C. coccineum* as a traditional wound healing remedy [71]. This effect has been ascribed to astringent and antihemorrhagic activity. However, one could assume that the efficacy (especially during old times) could be not only confined to stopping the bleeding, but could also synergistically involve the prevention of infections. Accordingly, antimicrobial activity of *C. coccineum* extracts has been investigated, as it would also be a favorable feature in nutraceutical or cosmetic formulations.

Aqueous extracts of specimens collected in Sardinia were tested using the disc diffusion method against several Gram-positive and -negative bacterial strains [28]. The extracts inhibited the growth of all the tested Gram-positive bacteria, including the clinical isolate MRSA *S. aureus* (methicillin-resistant). Despite the fact that anthocyanins have been reported to interfere with the permeability of bacterial membranes [72], Zucca and co-workers showed that antibacterial activity was not altered in the virtually anthocyanin-free extract obtained from the peeled plant. This observation suggests that the antibacterial activity of *C. coccineum* could be due to some other phytochemical (possibly gallic acid, the main component of these extracts; see Section 3). In fact, the organic acid and its derivatives (gallates) have been reported to have antimicrobial activity [73,74], probably due to their pro-oxidant effect. Unfortunately, these data have only been obtained using the disc diffusion method, and still need confirmation by more standardized approaches.

*C. coccineum* samples were also tested against some fungal species [50]. Supercritical fluid extraction (CO_2_–SFE) was used as an alternative to the classical systems of extraction with water or solvent to obtain the so-called fixed oil. This mixture of non-polar compounds thus obtained was compared with the plant methanolic extract. The fixed oil was less active than the latter, suggesting that antifungal activity could be due to some polar phytochemicals. The methanolic extract showed a Minimum Inhibitory Concentration (MIC) of 0.025 mg mL^−1^ against *Candida krusei*, *C. guilliermondii*, and *C. neoformans*, even lower than fluconazole, used as antifungal standard. The extract was, in general, active against *Candida* species, *Criptococcus neoformans*, and *Trichophyton* species.

Antifungal activity against *C. albicans* has been confirmed in the case of nanoformulations based on *C. coccineum* collected in Sardinia [51]. High speed stirring and high-pressure homogenization have been used to obtain almost uniform spherical particles in the nano range (3–600 nm). The authors showed that only the nanosamples obtained from the highly colored external layer inhibited the growth of the pathogenic fungus, whereas the colorless peeled plant was almost inactive, showing that different chemicals could be responsible for antifungal and antimicrobial activity.

## 6. Conclusions

Until now, studies providing an overview of *C. coccineum* have mostly emphasized its historical background without reviewing its chemical content and biological activities. In this work, we have drawn attention to this plant that, although known for many centuries, has been partly ignored by current phyto- and folk medicine. Ancient European and especially Arab medicine took *C. coccineum* into great consideration for the preparation of remedies against bleeding, diarrhea and dysentery, and disorders of the reproductive system.

As far as we know, this is the first review on *C. coccineum* that has taken stock of the information provided by the few scientific studies conducted on this plant in the last forty years, that is, since the interest in this plant began to be addressed with the modern tools of scientific investigation. These studies indicate many biological activities of *C. coccineum*, some already reported by traditional medicine, and some completely new. In summary, *C. coccineum* seems to possess antioxidant power, antitumoral activity, and effects on the reproductive and cardiovascular systems. Unfortunately, almost all the studies have been conducted in vitro, and only marginal in vivo confirmation has been done. In particular, antitumoral activity seems to be the most promising. By contrast, although very attractive, data about the plant’s effect on the cardiovascular system are still too limited. These facts, combined with its action against tyrosinase, suggest that it could be a good candidate for nutraceutical applications or as an additive in the food industry. Its antimicrobial activity, on the other hand, could open the way to cosmetic applications.

The effect on reproductive systems could also be very promising from a commercial perspective, but comparison with current drugs is still needed. Additionally, the exact chemical(s) responsible for this activity have still not been identified. This step could allow an understanding of whether the effect is topical or extends to central nervous system. In particular, this issue is still unresolved for the *C. songaricum*-based commercial preparations.

Only a few compounds have been identified in the plant so far, so further in-depth studies are needed to shed light on a plant for which the uses date back centuries, but which has not yet revealed all its potential. An extensive phytochemical characterization of the plant extracts seems to be a mandatory step, combined with deeper in vivo assays of their ability to counteract oxidative stress. While new ways of exploiting the medicinal properties of *C. coccineum* are being developed, it will become necessary to ensure that both subspecies can be protected from extinction, either by regulating their collection in the wild or by cultivating them, so that they can be used sustainably.

## Figures and Tables

**Table 1:** Some ancient uses of *C. coccineum* aerial part in folk medicine.

Folk Medical Uses	Geographical Area	Preparation	Ref.
Hemorrhoids	Mediterranean Basin	decoction	[18,19]
Dysentery; diarrhea	Mediterranean Basin	decoction; infused in white wine	[20,21,22]
Bleeding	Malta Island	dried up	[17]
Blood vomiting	Italy	decoction	[18,22]
Healing	Italy	dried up	[23]
Blood pressure	Italy	decoction	[18]
Venereal diseases	Mediterranean Basin	infusion	[17]
To strengthen teeth	Italy	powdered	[18,22]

**Table 2:** Main phytochemicals reported in *C. coccineum* extract.

Compound Class	Name	Extraction Method	Ref.
Flavonoids	Cyanidin 3-*O*-glucopyranoside	EtOH-H_2_O-HOAc; H_2_O	[14]
Flavonoids	Cyanidin 3-*O*-rhamnosylglucoside	not available	[25]
Organic acids	Gallic acid	H_2_O	[7]
Organic acids	Betaine	MetOH, and H_2_O	[33]
Organic acids	Bicarboxylic acids	MetOH, and H_2_O	[33]
Saccharides	Monosaccharides	MetOH, and H_2_O	[33]
Lipids	Lipid profile	Fixed oil	[33,34]
Lipids	Lipid profile	CHCl_3_:MetOH	[28]
Other compounds	Aminoacidic profile	MetOH, and H_2_O	[33]
Other compounds	Protein content	H_2_O	[28]

**Table 3:** Main antioxidant parameters reported for *C. coccineum* extracts.

Method of Extraction	Part of the Plant	[DPPH•] IC_50_ (µg·mL^−1^)	TPC (mg GA·g^−1^ of fde	FC (mg CE·g^−1^ of fde)	[ABTS•^+^] IC_50_ (µg·mL^−1^)	Ref.
EtOH	Aerial part	4.09 ± 0.6	406.38 ± 2	109.47 ± 33	n.a.	[27]
Water	Aerial part	13.47 ± 2.2	75.69 ± 3.6	39.19 ± 2	n.a.	[27]
MetOH	Whole plant (crude extract)	40.0	259.30 ± 6.8	d.n.q.	30.0	[5]
ButOH	Whole plant (crude extract)	5.60	201.36 ± 7.4	N.A.	6.0	[5]
MetOH	Aerial part	54.20 ± 2.1	173.50 ± 5.1	40.34 ± 0.7	910 ± 0.1	[7]
Water	Aerial part	51.60 ± 3.2	108.87 ± 3.7	37.15 ± 0.6	890 ± 0.1	[7]

TPC, total phenolic content; FC, flavonoid content; fde, freeze-dried extract; n.a., not available; GAE, gallic acid equivalent; CE, catechin equivalent; d.n.q., detected but not quantified.

**Table 4:** Main biological activities reported in *C. coccineum* extracts.

Biological Activity	The Parts of the Plant Used and Mode of Administration	Type of Study	Origin of the Plant	Ref.
(a) Antioxidant	Aerial part (ethanol extract)	in vitro	Algeria	[27]
	Whole plant (polar and non-polar extract)	in vitro	Saudi Arabia	[5]
	External layer (water extract)	in vitro	Italy (Sardinia)	[7]
	Aerial part (increasing polarity solvents extract)	in vitro	Italy; Tunisia	[33]
(b) Antimicrobial	Whole plant (methanol extract; fixed oil)	in vitro	Italy (Sardinia)	[50]
	Whole plant (nanoparticles preparation)	in vitro	Italy (Sardinia)	[51]
	Whole plant (water extract)	in vitro	Italy (Sardinia)	[28]
(c) Anti-tyrosinase	Whole plant	in vitro	Italy (Sardinia)	[28]
(d) Spermatogenesis and sperm motility	Inner pulp of the plant (water extract)	in vivo	Saudi Arabia	[4]
(d) Ovarian increase and folliculogenesis	Whole plant (water extract)	in vivo	Saudi Arabia	[6]
(e) Testicular development and spermatogenesis	Whole plant (water extract through a stomach tube)	in vivo	Saudi Arabia	[52]
(f) Pro-erectile	Aerial part (water extract subcutaneously)	in vivo	Italy (Sardinia)	[28]
(g) Inhibitory effect on the growth of colon cancer Caco-2 cells	Aerial plant (CO_2_ SFE)	in vitro	Italy (Sardinia)	[34]
(h) Inhibitory effect on the growth of B16F10 cells	Aerial part	in vitro	Italy (Sardinia)	[28]
(i) Anticancer	Whole plant fixed oil)	in vitro	Italy (Sardinia)	[37]
	Aerial part (water extract by peritoneal injection)	in vivo	Tunisia (Bizerte)	[53]
(l) Anti-tyrosinase	Whole plant	in vitro	Italy (Sardinia)	[28]
(m) Cardioprotective	Aerial part (methanol extract by oral administration)	in vivo	Saudi Arabia	[54]
(n) Blood pressure	Whole plant (fresh juice by oral administration)	in vivo	Iran	[55]
